# Varying modulation of HTLV-1 LTR activity by BAF complexes

**DOI:** 10.1186/1742-4690-8-S1-A180

**Published:** 2011-06-06

**Authors:** Rachel Van Duyne, Irene Guendel, Nazly Shafagati, Kylene Kehn-Hall, Rebecca Easley, Zachary Klase, Sergei Nekhai, Mudit Tyagi, Fatah Kashanchi

**Affiliations:** 1 George Mason University, Department of Molecular and Microbiology, National Center for Biodefense and Infectious Diseases, Manassas, VA, 20110, USA; 2 The George Washington University Medical Center, Department of Microbiology, Immunology, and Tropical Medicine, Washington, DC, 20037, USA; 3 National Institutes of Health, Molecular Virology Section, Laboratory of Molecular Microbiology, NIAID, Bethesda, Maryland, 20892, USA; 4 Howard University, Department of Microbiology, Center for Sickle Cell Disease, Department of Medicine, Washington DC, 20060, USA

## 

Chromatin remodeling is a rapidly emerging field with critical implications for the control of viral gene expression, especially for viruses with integrated genomes, such as HTLV-1. Recent observations indicate that there are as many as eight different BRG1 containing chromatin remodeling complexes highlight the advancement in the field, but also the necessity for future study especially when looking at viral infections. In the current study we focused on few of the Baf subunits that are common to most SWI/SNF complexes. We find that at least three Bafs, Baf53, Baf155 and Baf170, are highly regulated in HTLV-1 infected cells. Along these lines others have shown that depletion of Baf53 leads to the expansion of chromosome territories and decompaction of the chromatin. Here we show that there are clear varying differences between the Baf subunits after viral infection. These subunits also co-elute in different places from a sizing column and one particular form, Baf53 may be phosphorylated in HTLV-1 infected cells. Normally Baf53 is a suppressive complex and knock down experiments show increased level of virus gene expression from transfected or chronically infected cells.

